# AI Meets the Shopper: Psychosocial Factors in Ease of Use and Their Effect on E-Commerce Purchase Intention

**DOI:** 10.3390/bs14070616

**Published:** 2024-07-20

**Authors:** João M. Lopes, L. Filipe Silva, Ilda Massano-Cardoso

**Affiliations:** 1 Instituto Superior Miguel Torga, 3000-132 Coimbra, Portugal; ildamassano@ismt.pt; 2 NECE-UBI—Research Unit in Business Sciences, University of Beira Interior, 6201-001 Covilhã, Portugal; 3 Instituto Superior Miguel Torga & Instituto Superior de Contabilidade e Administração, Universidade de Aveiro, 3810-193 Aveiro, Portugal; filipersilva@ismt.pt; 4 Faculty of Medicine, University of Coimbra, 3000-548 Coimbra, Portugal; 5 CEISUC—Faculty of Economics, University of Coimbra, 3004-512 Coimbra, Portugal

**Keywords:** artificial intelligence, purchase intention, subjective norms, faith, consciousness, perceived control

## Abstract

The evolution of e-retail and the contribution of artificial intelligence in improving algorithms for greater customer engagement highlight the potential of these technologies to develop e-commerce further, making it more accessible and personalized to meet individual needs. This study aims to explore the psychosocial factors (subjective norms; faith; consciousness; perceived control) that affect AI-enabled ease of use and their impact on purchase intention in online retail. We will also assess the mediating effect of AI-enabled ease of use between psychosocial factors and consumer purchase intention. A quantitative methodology was used, and 1438 responses were collected from Portuguese consumers on e-retail. Structural equation modeling was used for the statistical treatment. The findings indicate that subjective norms do not positively impact AI-enabled ease of use, whereas factors such as faith, consciousness, and perceived control do enhance it. Furthermore, AI-enabled ease of use itself boosts purchase intention. Additionally, the effects of subjective norms, faith, consciousness, and perceived control on purchase intention are significantly enhanced when mediated by AI-enabled ease of use, highlighting the crucial role of usability in shaping consumer purchase behavior. The contribution of this study has been made through the formulation model that provides a systematized perspective about the influencers of purchase intentions and extends the knowledge about the impact of artificial intelligence in e-retail. Furthermore, this study offers insights into the impact of artificial intelligence in e-commerce—artificial intelligence directly affects purchase intentions and plays an important mediator role in the interaction mechanisms between psychosocial factors and purchase intentions.

## 1. Introduction

The future of e-commerce is being significantly shaped by rapid technological advances and the burgeoning field of artificial intelligence (AI). AI is at the forefront of transforming online retail by enhancing personalized shopping experiences, optimizing logistics, and creating more efficient supply chains [1]. AI, with its ability to analyze vast amounts of data and learn from shopping patterns, is not just revolutionizing the way businesses interact with consumers but also setting a new benchmark for operational efficiency. The shift towards decentralized platform development and the role of AI in refining algorithms for better customer engagement highlight the potential for these technologies to democratize e-retailing, making it more accessible and tailored to individual needs [2]. This technological leap forward promises to redefine the landscape of e-commerce, making it imperative for retailers to adapt to stay competitive.

Consumer behavior in the digital shopping realm is increasingly influenced by psychosocial factors, including subjective norms, faith, consciousness, consciousness, and perceived control, which in turn shape purchase intentions. The impact of AI-powered marketing tools on consumer behavior underscores the complex interplay between technology and psychology, where personalized marketing strategies are crafted based on AI analysis of a consumer’s online footprint, thereby subtly influencing their purchasing decisions [3]. The introduction of AI in online shopping has significantly altered consumer buying behavior, particularly through personalized product recommendations. Hallikainen et al. [4] highlight the profound impact AI has on enhancing the online shopping experience by delivering tailored suggestions to consumers based on their browsing and purchasing history. This personalized approach not only simplifies the decision-making process for consumers but also increases the likelihood of purchase decisions. The effectiveness of personalized recommendations can be attributed to their ability to (1) present products that align closely with consumer preferences and needs, (2) save consumers’ time by filtering out irrelevant options, and (3) increase the perceived value of the shopping experience through a bespoke selection. However, Canhoto et al. [5] notes potential downsides, such as the risk of overwhelming consumers with too many choices or the possibility of infringing on privacy. Nonetheless, the positive influence of personalized recommendations on purchase decisions outweighs these concerns, making it a valuable asset for online retailers aiming to enhance customer experience and loyalty [6].

AI-driven customer service, including chatbots and virtual assistants, has revolutionized the way online retailers interact with shoppers, significantly impacting shopper satisfaction. Over 92% of online shoppers have encountered AI customer service, and the quality of information provided by AI chatbots regarding product attributes is perceived to be superior to traditional methods [7,8]. This high level of satisfaction can be attributed to several factors: (1) immediate response to customer inquiries, reducing wait times; (2) availability 24/7, offering assistance at any time; (3) personalized interaction, adapting responses based on the customer’s shopping behavior and preferences. Ameen et al. [9] further elaborate that the convenience and efficiency provided by AI customer service translate into a competitive advantage for online retailers, as it significantly enhances the overall shopping experience. The continuous advancements in AI technology further promise to elevate customer satisfaction, making it a crucial element for success in the competitive online retail landscape [10].

While AI technologies offer numerous advantages in online shopping, they also raise significant concerns regarding consumer trust and privacy. Nearly 82% of consumers express considerable worry about how their data is utilized in AI-driven marketing, underlining the importance of data privacy in earning consumer trust [11]. These concerns stem from the following factors: (1) the opaque nature of AI algorithms, leaving consumers uncertain about how their information is used; (2) potential misuse of personal data without explicit consent; and (3) the risk of data breaches exposing sensitive information. Despite these concerns, studies suggest that establishing clear, transparent policies regarding AI data usage and implementing stringent security measures can mitigate these fears, thereby preserving consumer trust and loyalty [12,13]. Furthermore, understanding and addressing the drivers of consumer trust can significantly influence online shopping behaviors, suggesting that retailers who prioritize privacy and transparency in their AI initiatives can maintain a strong relationship with their customers [14].

Psychosocial factors (subjective norms; faith; consciousness; perceived control) play a critical role in shaping consumer behavior towards AI-enabled services in online retail environments. Regarding subjective norms, this phenomenon is supported by research indicating that subjective norms significantly influence behavioral intentions by enhancing perceived usefulness and ease of use [15]. For instance, if an individual sees their friends frequently using AI-powered recommendation systems to make purchasing decisions, they may feel more inclined to trust and utilize similar technologies. Faith, or the degree of trust individuals place in AI technologies, can substantially impact their willingness to engage with these systems. Kelly et al. [16] have shown that higher levels of trust correlate with increased acceptance and usage of AI tools. On the other hand, consciousness, which pertains to an individual’s awareness and consideration of ethical implications, can either facilitate or hinder the adoption of AI technologies [17]. For example, consumers who are conscious of privacy concerns may be hesitant to use AI services that require extensive data sharing. Perceived control refers to the extent to which individuals believe they have control over the AI technologies they use. Ye, Xue, He, Gu, Lin, Xu, and Cheng [15] highlight that perceived behavioral control can indirectly influence the intention to use AI services by affecting perceived ease of use. When consumers feel they can easily navigate and customize AI tools according to their preferences, they are more likely to perceive these technologies as beneficial and user-friendly. For instance, an AI-driven recommendation engine that allows users to adjust settings and filter results according to their criteria can enhance their sense of control, thereby increasing their satisfaction and likelihood of continued use [18]. Hence, improving perceived control can be a strategic approach for online retailers to foster greater acceptance of AI-enabled ease among their customers.

The intersection of AI and psychosocial factors in online retail is a critical but underexplored research area. Despite the rapid adoption of AI technologies in the retail sector, comprehensive studies examining how these technologies interact with human psychological and social behaviors are conspicuously absent. This gap is significant as understanding the intricate interplay between AI and psychosocial factors is crucial for developing AI-enabled platforms that meet consumer needs and preferences effectively. The literature indicates a recognition of the need to explore this intersection, yet there is a lack of systematic efforts to address this research void. This deficiency in focused academic attention hampers the ability of retailers to fully leverage AI technologies to enhance the online shopping experience, highlighting a pressing need for dedicated studies in this area [19,20]. On the other hand, existing studies tend to focus more on the technical capabilities and performance metrics of AI tools rather than their psychological impact on consumers, leaving a significant void in understanding how these technologies can be optimized for positive consumer experiences and long-term customer relationships [21,22]. Additionally, there is limited research directly correlating AI-enabled platforms with purchase intentions, despite general acknowledgment that user-friendly AI features can enhance the online shopping experience. The lack of empirical evidence linking the ease of use of AI technology to purchase intentions poses a problem for online retailers who aim to invest in AI to drive sales and improve customer loyalty. Addressing this research gap could offer valuable insights for retailers looking to use AI for competitive advantage, indicating a need for targeted studies that specifically investigate the connection between AI-enabled ease and consumer purchase decisions [16,23]. That said, it is important to answer two questions: What psychosocial factors affect AI-enabled ease of usage? Does AI-enabled ease of usage to affect purchase intention?

The present study aims to explore the psychosocial factors that affect AI-enabled ease of usage and its impact on purchase intention in online retail. We will also assess the mediating effect of AI-enabled ease of use between consumers’ psychosocial factors and purchase intentions.

This study makes five important contributions. Firstly, it provides a systematic framework for understanding the impact of AI-enabled ease of use on consumer purchase intentions in e-retail, highlighting its direct effect and mediating role in influencing psychosocial factors. This demonstrates the potential of AI to enhance consumer engagement and simplify shopping processes. Secondly, it explores the interaction between AI-enabled ease of use and psychosocial factors, noting that elements such as consciousness significantly affect AI adoption, thus suggesting that AI systems should be user-friendly to foster confidence. Thirdly, it emphasizes the role of subjective norms in predicting the adoption of AI for online purchases, showing that societal influences affect technology acceptance and purchase intentions. Fourthly, it validates that higher consumer satisfaction and confidence in AI promote a greater willingness to adopt AI in e-retail, thereby enhancing purchase intentions. Finally, the research underscores AI potential in e-retail marketing to boost consumer engagement, satisfaction, loyalty, and sales, offering companies a valuable tool for building stronger consumer relationships and driving sales growth.

## 2. Literature Review

### 2.1. Technology Acceptance Model and the Theory of Planned Behavior

Combining the Technology Acceptance Model (TAM) and the Theory of Planned Behavior (TPB) provides a holistic approach to understanding AI adoption in e-retailing. The integration of these models allows for a more comprehensive analysis by considering both technological and psychosocial factors. TAM emphasizes perceived ease of use and perceived usefulness as primary predictors of technology acceptance [24], while TPB incorporates attitudes, subjective norms, and perceived behavioral control [25]. By merging these frameworks, researchers can better predict and explain user behavior toward AI in e-retailing. For example, an integrated model might reveal that while users find AI tools useful and easy to use, their adoption is also heavily influenced by social norms and their confidence in managing these tools. This combined approach not only enhances the predictive power of the models but also provides deeper insights into the multifaceted nature of AI adoption.

For this study, we considered subjective norms, faith, conscientiousness, and perceived control as psychosocial factors). Subjective norms significantly influence attitudes toward AI adoption in e-retailing by shaping individual perceptions of social pressure and expectations. According to the TPB, subjective norms are the perceived social pressures to perform or not perform a particular behavior [26]. In the context of e-retailing, if influential groups such as peers, family, or industry leaders advocate for the use of AI technologies, individuals are more likely to develop favorable attitudes toward adopting these systems. Sohn and Kwon [27] indicate that attitudes, subjective norms, and perceived behavioral control collectively predict behavioral intentions, thereby linking social influences with technology acceptance. Faith plays a crucial role in building trust and belief in AI systems for e-retailing, which in turn affects user acceptance. Trust is a fundamental component in the TAM, influencing perceived usefulness and perceived ease of use, which are central to the model [15]. In e-retailing, faith in the reliability and security of AI systems can alleviate user concerns about data privacy and accuracy. Kelly, Kaye, and Oviedo-Trespalacios [16] have shown that trust significantly predicts behavioral intention and willingness to use AI technologies. Consciousness regarding AI awareness and ethical considerations is increasingly important in the utilization of AI in e-retailing. Awareness of AI capabilities and limitations can shape user perceptions of its usefulness and ease of use, which are critical factors in the TAM [28]. Ethical considerations, such as data privacy, algorithmic bias, and transparency, are also integral to user acceptance. As consumers become more informed and conscious of these issues, their adoption decisions will be influenced by how well e-retailers address ethical concerns. The integration of ethical AI practices can thus build consumer trust and positively impact the perceived control over using these technologies [27]. Perceived control is a crucial element in understanding user confidence in managing AI tools within the e-retailing sector. This concept, derived from the TPB, suggests that when users feel they have control over the AI tools they are using, their likelihood of adopting and utilizing these technologies increases. For instance, perceived usefulness and performance expectancy have been shown to significantly and positively predict behavioral intentions toward AI adoption [16]. Thus, behavioral intentions in AI usage can be predicted by integrating various psychosocial factors, such as subjective norms, faith, consciousness, and perceived control, with the TAM and the TPB.

### 2.2. Artificial Intelligence in E-Retailing and Consumption

The integration of AI in e-retailing has revolutionized the way shopping experiences are personalized for consumers. Through the utilization of AI-powered algorithms and machine learning, retailers can now tailor recommendations and content to align closely with each customer’s unique preferences and past purchasing behaviors [29]. This level of personalization not only enhances the shopping experience but also fosters a deeper connection between consumers and brands. Machine learning, in particular, plays a pivotal role by analyzing vast amounts of data to identify patterns and preferences, thus enabling a more targeted and efficient shopping journey [30]. The significance of these AI-driven personalization efforts is underscored by their ability to create a seamless and highly individualized shopping environment, which is instrumental in driving customer satisfaction and loyalty [2].

AI-driven customer service solutions are another cornerstone in enhancing customer satisfaction within the e-retailing sector. These solutions, ranging from chatbots to virtual assistants, leverage AI’s capabilities to offer timely and relevant support to customers [31]. The impact of these AI-powered service options is profound, as they not only provide instant assistance but are also capable of learning from interactions to improve future responses. This continuous improvement loop ensures that customer service experiences become more personalized and effective over time. Furthermore, the implementation of AI in customer service aligns with the evolving expectations of consumers for quick, efficient, and accurate support, thereby significantly contributing to overall customer satisfaction [32]. The ability of AI to manage and resolve customer inquiries with such precision and speed exemplifies its critical role in modern customer service strategies.

Predictive analytics powered by AI is transforming how product recommendations and search results are optimized in e-retailing. By analyzing historical data, current trends, and consumer behavior, machine learning algorithms excel at forecasting future purchasing patterns and preferences [33,34]. This predictive capability enables retailers to proactively offer products and services that consumers are more likely to be interested in, thereby enhancing the relevance and effectiveness of recommendations. The precision of these predictive analytics is a testament to the advanced capabilities of AI, which can sift through and analyze large datasets to identify meaningful insights and trends [35]. This not only improves the accuracy of product recommendations but also optimizes search results to match closely with consumer intentions and interests. As a result, predictive analytics serves as a powerful tool in curating a more personalized and satisfactory shopping experience, further emphasizing the instrumental role of AI in shaping the future of e-retailing [36].

AI is transforming e-retailing, not just with its recommendation engines and dynamic pricing but also by creating a smoother and more user-friendly experience [37]. This AI-enabled ease of use can significantly impact consumer behavior. Psychosocial factors, like subjective norms (feeling you should shop online), faith in the technology, and a sense of control over the process, all influence purchase intention. By simplifying the online shopping journey, AI could address these factors, making e-commerce more appealing and the intention to buy more likely [38].

### 2.3. Artificial Intelligence-Enabled Ease of Use

Defining enabled ease of use in the context of AI involves understanding how AI systems are designed to be user-friendly, allowing users to interact with and benefit from AI technology without needing extensive technical knowledge [18]. This concept is crucial in the development and deployment of AI solutions, as it directly impacts user adoption and satisfaction. The term enabled ease of use refers to the seamless integration of AI into everyday applications, making complex processes appear simple and intuitive to the end-user. The importance of user-friendly AI systems in technology adoption cannot be overstated. The ease of use significantly affects a user’s willingness to engage with and adopt new technologies [39]. In the context of AI, this means that systems designed with the user in mind, prioritizing straightforward interaction and accessibility, are far more likely to be embraced by the target audience. This user-friendly approach not only enhances the user experience but also encourages positive outcomes such as increased purchase intentions and customer loyalty [40]. Bhagat et al. [41] demonstrated that AI-enabled ease of use simplifies the consumer’s purchase intention, making it easier for them to navigate e-retailing platforms and thereby boosting their likelihood of making a purchase. These findings highlight the direct link between the ease of use of AI systems and their effective adoption and success in the market.

Achieving ease of use in AI solutions presents several challenges, primarily due to the complex nature of AI technology itself [42]. Designing AI systems that are both powerful and user-friendly requires a delicate balance between technical sophistication and simplicity. Challenges include ensuring AI systems can interpret user input accurately, providing real-time assistance without overwhelming the user, and maintaining user privacy and security [43,44]. Additionally, the AI needs to continuously learn and adapt to cater to evolving user needs and preferences.

### 2.4. Psychosocial Factors

#### 2.4.1. Subjective Norms

Understanding subjective norms within the context of technology adoption requires a foundational grasp of how societal expectations and peer influences shape individual behaviors toward new technologies. The theory of planned behavior highlights that subjective norms—individuals’ perceptions of whether people important to them support the use of technology—are pivotal in determining their intention to adopt it [25]. The subjective norms, encompassing the influence of peers, colleagues, and societal trends, play a critical role in forming individuals’ attitudes toward AI-enabled technologies [28]. These attitudes then significantly impact their perceived ease of use and, ultimately, their satisfaction with and acceptance of the technology [45]. For instance, in environments where AI adoption is encouraged and valued, individuals are more likely to perceive these technologies as useful and easy to use, thereby fostering a more accepting attitude toward their integration into daily tasks [18]. This phenomenon underscores the importance of social context and the prevailing norms within it as key determinants of AI technology’s perceived usability and acceptance.

Analyzing empirical evidence linking subjective norms to enhanced ease of use in AI applications reveals a nuanced relationship. While some studies affirm a strong positive connection between subjective norms and perceived ease of use [46], others suggest a more complex interaction, with perceived usefulness acting as a mediator in this relationship [18]. For instance, research involving AI-enabled large language models (LLM) indicates that subjective norms significantly influence their perceived ease of use, highlighting the role of social endorsement in technology adoption [47]. However, the relationship between subjective norms and technology acceptance is not always straightforward, as evidenced by studies reporting a weak correlation between social norms and perceived usefulness [48]. This discrepancy points to the need for a deeper investigation into how subjective norms interact with other factors, such as personal attitudes and the inherent characteristics of the technology, to influence its perceived ease of use and overall acceptance.

**H1:**  *Subjective norms favorably affect AI-enabled ease of use*.

#### 2.4.2. Faith

Understanding the concept of faith in technology adoption begins with recognizing the multifaceted nature of faith itself. In general, faith is broadly understood as a form of basic knowledge that is intertwined with a certainty that leaves no room for doubt. This conceptualization of faith highlights its role beyond mere cognitive processes, extending into the realm of emotional and psychological security [49]. Faith, in the context of technology, can be seen as trust or confidence in the capabilities and reliability of technological solutions [50], including AI. This form of faith is crucial for the initial adoption and continued use of technology solutions. It influences how individuals perceive and interact with technology on a daily basis. Faith significantly affects the ease of use of AI-enabled tools, suggesting that when users have faith in technology, they are more likely to find it easy to use [41]. This is particularly relevant in today’s rapidly evolving digital landscape, where AI is becoming increasingly integrated into everyday life, raising questions and discussions about its impact and the trust we place in it [12,51].

The impact of faith on user perceptions of AI ease of use is substantial. Users who exhibit a higher level of faith in AI technology are more likely to perceive it as easy to use. This perception is crucial for the widespread adoption and effective utilization of AI technologies in various sectors. Jain et al. [52] delineated how ease of use, a key component of technology acceptance models, is significantly influenced by the user’s faith in the technology. For instance, in the realm of online consumer behavior, faith in AI has been linked to a more favorable perception of AI-enabled ease of use, which in turn affects consumers’ online shopping behaviors and their acceptance of AI recommendations [41]. Furthermore, understanding the determinants of perceived ease of use, including faith, can enable domain-independent progress in AI research and application, thereby enhancing user experience across different interfaces and platforms.

Studies in various sectors demonstrate faith’s significant role in enhancing AI interface interactions. For example, in education, teachers’ perceptions of AI for social good and their self-efficacy in learning AI technologies are directly influenced by their faith in the technology’s potential and capabilities [53]. This faith not only impacts their comfort and satisfaction with using AI tools but also predicts changes in these attitudes over time [54]. In the religious domain, the integration of AI has prompted discussions on how faith intersects with technology, influencing both the acceptance of AI and its perceived usefulness in supporting religious practices and experiences [55]. These case studies underscore the importance of faith in technology adoption, particularly in AI, where ease of use and user interface interactions are pivotal for successful integration and beneficial outcomes.

**H2:**  *Faith favorably affects AI-enabled ease of use*.

#### 2.4.3. Consciousness

Exploring the concept of human consciousness reveals its profound significance in the advancement of AI systems aimed at user-friendliness. Human consciousness, a complex phenomenon without a universally accepted definition, introduces computational challenges and opportunities in AI development [56]. This exploration is not merely academic, it has practical implications for designing AI systems that are intuitive and easy to use. By understanding consciousness, AI developers can create systems that better mimic human thought processes, potentially making these systems more relatable and easier for users to interact with. The significance of consciousness in AI usability lies in its potential to bridge the gap between human cognitive processes and machine operations, thereby enhancing the natural interaction between humans and machines [57]. As we delve into the computational aspects of consciousness, we unlock new possibilities for creating AI that not only performs tasks but does so in a way that feels more natural and intuitive to human users. For instance, Kim and Im [58] demonstrated that users tend to anthropomorphize AI, attributing human-like qualities to these systems, which can significantly affect their perceptions of the AI’s capabilities and ease of use. Furthermore, incorporating aspects such as humanoid appearance and social capabilities in retail service robots has been shown to positively influence consumer attitudes towards human–robot interaction [59]. By understanding and integrating elements of human consciousness into AI design, developers can create systems that are not only more efficient but also more aligned with human user expectations, thereby enhancing the overall user experience. AI systems that can understand and predict user needs and preferences by mimicking aspects of human consciousness could revolutionize the way we interact with technology [57]. Additionally, as users come to perceive AI systems as more conscious or human-like, their trust in and satisfaction with these systems is likely to increase, further enhancing user experience [60].

**H3:**  *Consciousness favorably affects AI-enabled ease of use*.

#### 2.4.4. Perceived Control

Understanding perceived control within the context of AI involves recognizing the user’s belief in their ability to interact with and influence AI-enabled systems effectively. This concept is crucial as it directly affects the user’s confidence and willingness to engage with AI technologies. According to Esmaeilzadeh [61], when users feel they have greater control over AI, their mistrust and perceived risks associated with AI mechanisms decrease. This sense of control can stem from various factors, including the user’s familiarity with the technology, the transparency of AI operations, and the degree to which users can customize their interactions with AI systems. Essentially, perceived control acts as a bridge between the user and complex AI technologies, mitigating fears and enhancing trust.

In this context, users who perceive a higher level of control over an AI system are more likely to explore its full potential and integrate it into their daily tasks. This enhanced interaction is often fueled by a reduction in perceived risk and an increase in trust in the technology [51]. Furthermore, perceived control encourages users to experiment and learn, leading to a deeper understanding of the AI system’s capabilities and limitations. When users feel in control of AI, they are more likely to find the system easy to use, which encourages further use and acceptance of the technology [62]. As a result, users become more competent in exploiting AI functionalities, which, in turn, improves their overall experience and satisfaction with the technology. This positive feedback loop is critical for the successful adoption of AI systems, as it not only enhances user satisfaction but also contributes, thereby fostering a more widespread acceptance and integration of AI into everyday activities [63].

**H4:**  *Perceived control favorably affects AI-enabled ease of use*.

#### 2.4.5. Purchase Intention

AI technologies have been instrumental in simplifying the shopping experience by personalizing user interactions, optimizing search functionalities, and providing virtual assistant services. This has made navigating e-commerce websites more intuitive, significantly reducing the effort required to find and purchase products [37]. In this context, purchase intention refers to a consumer’s plan or decision to buy a product or service. The ease of use enabled by AI directly influences this intention by enhancing the overall shopping experience, making it more convenient and less time-consuming for the customer [64].

The usability and accessibility of online platforms highly influence consumer behavior in the digital marketplace. AI-enabled features such as chatbots for instant customer service, recommendation engines for personalized product suggestions, and voice search for hands-free navigation play a crucial role in enhancing these aspects [2,65]. Constantinides [66] showed that these features significantly impact user satisfaction by catering to individual needs and preferences, thereby making the online shopping experience more engaging and less overwhelming. A positive response from consumers is expected towards these AI-enabled enhancements to improve their effectiveness, usability, and accessibility.

The correlation between AI-enabled ease of use and purchase intention among consumers is strongly supported by empirical evidence. Improved ease of use due to AI not only enhances the shopping experience but also positively affects the likelihood of consumers making a purchase. This is because a more user-friendly and accessible platform reduces the cognitive load on shoppers, making them more inclined to proceed with their purchase decisions [66]. Furthermore, the enhanced interaction with AI features creates a more personalized and enjoyable shopping journey, which increases customer satisfaction and loyalty [32]. These factors combine to forge a direct link between the convenience offered by AI and the willingness of consumers to engage in transactions, thereby highlighting the pivotal role of AI in driving online purchase intentions.

**H5:**  AI-enabled ease of use positively affects purchase intention.

### 2.5. The Mediating Effect of AI-Enabled Ease of Use

The mediating effect of AI-enabled ease of use on the relationship between subjective consumer norms and purchase intention is a testament to the transformative power of technology in modern commerce [67]. AI’s ability to interpret and respond to consumer preferences while providing a seamless and effortless user experience acts as a bridge between social influences and the final decision to purchase. This mediating role underscores the complexity of the decision-making process, where AI-enabled ease of use translates social expectations and norms into positive attitudes toward products, ultimately leading to a higher likelihood of purchase intention [68]. The interaction between subjective norms, AI-enhanced ease of use, and purchase intention highlights a nuanced pathway through which consumers navigate their choices, influenced by both social circles and technological advancements.

**H6:**  *Consumers’ subjective norms positively affect purchase intention when mediated by AI-enabled ease of use*.

The role of AI-enabled ease of use in mediating the relationship between consumer faith and purchase intention is significant. When AI technologies are perceived as easy to use, this perception enhances the positive impact of consumer faith on their intention to purchase [69]. This mediation effect can be understood through the lens of the Technology Acceptance Model, which posits that the perceived ease of use of a technology not only directly affects the intention to use that technology but also amplifies the effect of perceived usefulness [69]. Acikgoz et al. [70] found that consumers’ engagement with AI-powered voice assistants positively influenced their purchase intentions, mediated by perceived ease of use and usefulness. In essence, if a consumer has faith in AI technology and find it easy to use, they are more likely to perceive it as useful, which in turn positively influences their purchase intentions.

**H7:**  *Consumer faith positively affects purchase intention when mediated by AI-enabled ease of use*.

The mediating effects of AI-enabled ease of use on the relationship between consumer consciousness and purchase intention are profound and multifaceted. AI technologies not only enhance the ease with which consumers can navigate the shopping environment but also play a crucial role in aligning with the dimensions of consumer consciousness to influence purchase intentions positively: (1) By offering personalized recommendations, AI aligns with the consumer’s quality sensitivity and brand consciousness, making it easier for consumers to find products that meet their specific criteria. (2) The convenience and efficiency provided by AI technologies address price awareness by helping consumers quickly identify the best-value options, thereby enhancing the perceived ease of use and positively influencing purchase intentions [71]. (3) Augmented reality and virtual try-ons cater to ethical awareness by allowing consumers to make more informed decisions, thereby reducing the likelihood of returns and the environmental impact associated with them [72]. (4) The overall enhancement of the shopping experience through AI-driven technologies fosters a positive attitude towards technology, further strengthening the link between consumer consciousness and purchase intention by making the process more intuitive and satisfying [69]. In summary, the mediating role of AI-enabled ease of use embodies a crucial bridge between consumer consciousness and purchase intention, facilitating a more seamless, efficient, and personalized shopping experience that aligns with consumer preferences and expectations.

**H8:**  *Consumer conscientiousness positively affects purchase intention when mediated by AI-enabled ease of use*.

The advent of AI in online shopping platforms has introduced a new dimension to how perceived control can influence purchase intention [73]. AI-enabled ease of use acts as a critical mediator in this relationship, enhancing the consumer’s shopping experience by making navigation, information retrieval, and decision-making processes more intuitive and less effort-intensive. The mediation occurs as AI technologies personalize the shopping experience, providing recommendations and assistance tailored to the consumer’s preferences and past behaviors. This personalization, in turn, enhances the user’s perceived control over the shopping process by making it seem more manageable and aligned with their needs and desires. The seamless integration of AI not only simplifies the decision-making process but also significantly reduces the effort required to make informed decisions, thereby positively influencing the consumer’s intention to purchase through enhanced perceived control and ease of use [62,74].

**H9:**  *Consumers’ perceived control positively affects purchase intention when mediated by AI-enabled ease of use*.

**Figure 1:**
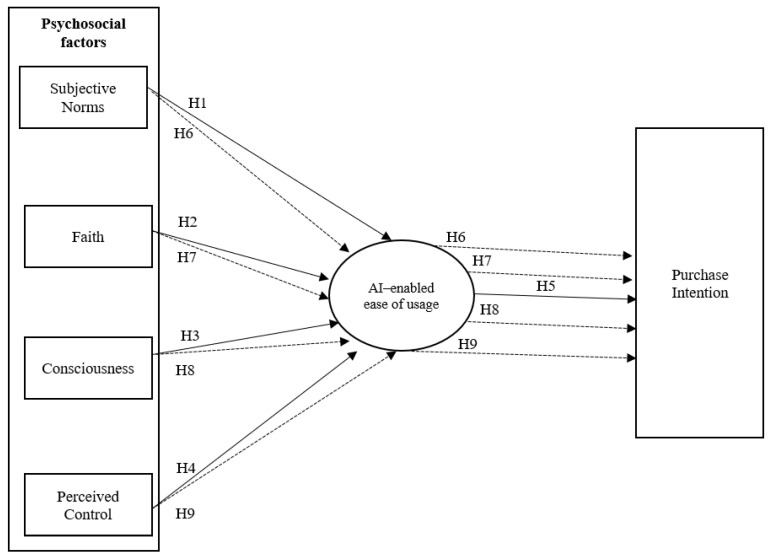
Research model. Note: direct effects (→) and mediating effects (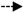).

In Figure 1, the research model was formulated.

## 3. Method

### 3.1. Participants

A cross-sectional study was conducted using a convenience sample encompassing 1438 subjects (798 women, 55.5%, 625 men, 43.5%, 5 nonbinary genders, 0.3%, and 10 preferred not to answer, 0.7%). The mean age was 27.78 (*SD* = 12.84), ranging from 18 to 88 years old. Concerning years of education, 85 (5.9%) participants completed nine years or less of education, 568 (39.5%) completed secondary school, 640 (44.5%) had a university graduate degree, 115 (8%) had a master’s degree, and 30 (2.1%) had a doctoral degree.

### 3.2. Instruments

A sociodemographic questionnaire was used to describe the participants’ demographic characteristics. Table 1 shows the instruments used in this research, their definition, and the previous studies from which they were adapted.

All items are answered using a 7-point Likert scale, ranging from *strongly disagree* (1) to *strongly agree* (7) (e.g., SN: “People around me prefer me to shop from AI-enabled sites?”; F: “I prefer AI-enabled e-retailing.”; C: “I possess knowledge about the use of AI applications.”; PC: “When browsing this shopping website, I can control the virtual interactions according to my choices.”; AI: “AI-enabled shopping increases efficiency”; PI: “I am willing to spend more on purchases through online stores that are powered by AI technology”). The PI and AI dimensions were defined as the dependent variables, and the other dimensions (subjective norms, faith, consciousness, and perceived control) were defined as independent variables. In the current study, Cronbach’s alpha values of these dimensions ranged from 0.54 (PC) to 0.84 (AI).

### 3.3. Procedures

The study protocol was approved by the Ethics Committee. The original items suffered a process of translation from English to European Portuguese, followed by back-translation to confirm the content correspondence [85]. The translation was completed by the research team, and a native speaker performed the back-translation. To further assess the items’ intelligibility and comprehensibility, a pilot study was conducted with 20 Portuguese consumers. These participants reported no difficulties in understanding the items’ content. The guidelines of the International Test Commission (2017) were adhered to throughout the study. The research was disseminated through social media platforms such as WhatsApp, Facebook, and Instagram, with a link provided on the Google Forms platform for accessing the study protocol. All participants were briefed on the study’s objectives. Their participation was voluntary. Informed consent was mandatory, and measures were taken to ensure the anonymity and confidentiality of the collected data. Data collection occurred between September 2023 and January 2024.

### 3.4. Statistical Analysis

For data analyses, the JASP software package version 0.16.4 (JASP Team, 2022) and IBM Software Statistical Package for Social Sciences (SPSS v.28) (IBM Corp. Released, 2022) were used.

Descriptive statistical analysis was performed for the quantitative sociodemographic variables using mean and standard deviation and using frequencies and percentages for nominal variables. 

The model proposed has been developed and tested through SEM to measure the constructs, obtained from the path model using the following goodness-of-fit indices: CMIN/*df* (considering values ranging from 1 to 2 a good fit); the Comparative Fit Index (CFI); the Tucker and Lewis Index (TLI) (CFI and TLI values between 0.90 and 0.95 represent a good fit); and the Standardized Root Mean Square Residual (SRMR) (values < 0.08 determine an acceptable fit) [86]. The Root Mean Square Error of Approximation (RMSEA) was also used (90% confidence intervals, values between 0.05 and 0.10 suggesting a good fit) [87]. 

The Pearson correlation coefficient was calculated to estimate convergent validity. To assess discriminant validity, the average variance extracted (AVE) was estimated [88]. AVE should be greater than 0.5. To test the reliability of each dimension, Cronbach alpha was computed as well as composite reliability (CR). According to Hair et al. [89], composite reliability (CR) must be above 0.7. The independent samples t-test was used to compare means between groups. Effect size was calculated based on Cohen’s *d* effect sizes (*d* = 0.01 is considered very small, *d* = 0.20 is small, *d* = 0.50 is medium, *d* = 0.80 is large, *d* = 1.20 is very large, and *d* = 2.00 is huge) Sawilowsky [90]. Pearson correlations were calculated between dimensions and considered as small (*r* = 0.10 to 0.29), moderate (*r* = 0.30 to 0.49), large (*r* = 0.50 to 0.69), very large (*r* = 0.70 to 0.89), nearly perfect (*r* ≥ 0.90), and perfect (*r* = 1) [91]. A multiple linear regression was conducted considering artificial intelligence (AI) and intention purchase (IP) dependent variables for the regression analysis. Thus, the absence of multicollinearity was confirmed by the calculation of the Variance Inflation Factor (VIF) (VIF < 5) [92].

To examine mediator effects of AI in IP, a path analysis was conducted by structural equation modeling (SEM) [93,94]. To assess the significance of the mediational paths, a bootstrap resampling approach was used with 5000 bootstrap samples and 95% confidence intervals (CIs) [94]. The significance level used for all analyses was <0.05.

## 4. Results

According to the model presented in Figure 1, the model was tested through these fit measures: CMIN/*df* = 5.74; χ^2^_(137)_ = 786.75, *p* < 0.001; CFI = 0.95; TLI = 0.94; RMSEA = 0.06 [0.05–0.06]; SRMR = 0.04. Standardized factor loadings, Cronbach’s alpha, CR, and AVE were computed. To estimate the reliability of each dimension, Cronbach’s alpha was calculated. Values found for the different dimensions were as follows: SN = 0.82; F = 0.82; C = 0.69; PC = 0.57; AI = 0.84; and PI = 0.81. The AVE results ranged from 0.39 (PC) to 0.64 (AI) (C). Composite reliability ranged from 0.70 to 0.83. The results show that the average variance extracted (AVE) is not greater than 0.5 to the perceived control (PC) dimension. Indeed, composite reliability (CR) was equal to or above 0.7. for all dimensions that indicate the data used in the study to be reliable and valid (Table 2).

Table 3 presents the correlations between all dimensions, and all were statistically significant. The correlational results indicate the concurrent validity of the measures used. Correlations were positive between AI and PC (*r* = 0.527; *p* < 0.001), C (*r* = 0.687; *p* < 0.001), F (*r* = 0.671; *p* < 0.001), SN (*r* = 0.514; *p* < 0.001), and PI (*r* = 0.609; *p* < 0.001). The variance inflation factor (VIF) analysis was calculated, and values ranged from 1.61 (PC) to 2.26 (F). The fact that all VIF values are below 5 suggests that a multiple regression model can proceed without significant multicollinearity issues.

After validating the fulfillment of assumptions, including adequate sample size and addressing multicollinearity, a multiple linear regression analysis was conducted to explore the predictors of AI. The resulting model accounted for 58% of the total variance of the AI (Table 4). The results tested hypotheses H1, H2, H3, and H4, with H1 being rejected.

To test if artificial intelligence-enabled ease of usage (AI) positively affects purchase intention, as postulated by H5, another model was conducted through linear regression analysis (Table 5).

The results suggest that three independent variables were significant predictors of the dependent variable (AI): faith (F) (ß = 0.34, *p* < 0.001); consciousness (C) (ß = 0.40, *p* < 0.001), and perceived control (PC) (ß = 0.12, *p* < 0.001). The artificial intelligence-enabled ease of use (AI) was evidenced as being predictive of purchase intention (PI).

To test others hypotheses (H6, H7, H8, H9), a bootstrap analysis was computed. Table 6 displays the results of estimating indirect relationships between constructs established in the research model (Figure 1). All relationships are statistically significant.

Subjective norms (SN), faith (F), consciousness (C), and perceived control (PC), when mediated by artificial intelligence-enabled ease of use (AI), positively influence the purchase intention (PI) (*β* = 0.195; *p* < 0.001; *β* = 0.195; *p* < 0.001; *β* = 0.299; *p* < 0.001; *β* = 0.227; *p* < 0.001).

## 5. Discussion of Results and Implications

### 5.1. Discussion of Results

Artificial intelligence has transformed the way that economic agents carry out business, particularly the relationship between companies and consumers. Due to the relevance of this theme, this study aimed to explore the influence of the psychosocial factors—subjective norms (SN), faith (F), consciousness (C), and perceived control (PC)—on the practical applicability of artificial intelligence-enabled ease of usage (AI) and its impact on purchase intention (PI) in online retail.

The study, based on the quantitative method, was conducted by a research model to observe the significance of the nine hypotheses that represent the influence and the impact they induce in PI. The results of this study show the following significant direct and indirect effects: faith (F), consciousness (C), and perceived control (PC) are the direct influencers of AI. AI positively influences PI in online retail and plays a mediator role in the psychosocial factors’ interaction mechanisms with PI. These results have implications for the Theory of Planned Behavior (TPB) and the Technology Acceptance Model (TAM).

Regarding the direct effects observed, our findings demonstrated, first, that SN does not predict AI directly. Consequently, hypothesis H1 is not validated, as can be confirmed by the multiple linear regression analysis—low statistical significance. These results contradict prior studies [25,28,46] by demonstrating that SN does not influence AI, which means that the beliefs and collective opinions of friends, family, and the general community do not have an impact on AI. Hence, we cannot affirm that social orientations or social pressures influence or motivate users to opt for AI experiences. This evidence complements the TPB [25] regarding the role that SN plays in AI acceptance, extending the knowledge about users’ behavior toward AI.

On the other hand, it was observed that F positively influences AI, so hypothesis H2 is validated. These findings corroborated prior studies [41,51,53] and reinforced the existing conception of the important role that F plays in AI experiences. AI services can improve the faith of users when providing a desired virtual expertise [41]. In accordance, confidence, reliability, and ease of use are valid arguments to stimulate the option of AI tools [41,52,55], which companies must understand to develop services that provide highly convenient, reliable, and beneficial virtual experiences. Hence, for companies, AI can be an opportunity to generate reliable business and also constitutes one (virtual) partner to increase engagement with consumers.

Next, this study found that C is an important factor that influences AI, which is consistent with the findings of previous studies [57,58,59] and validated hypothesis H3. This predictive result means that, generally, users feel safer and confident when they decide to opt for AI tools that offer intuitive interaction performance that positively affects their perceptions about the AI’s capabilities and ease of use [58]. Hence, the experience with AI can be great when the users’ consciousness about their expectations and intentions has been satisfied through AI technologies. Consequently, companies should provide transparent information about their offer and easy-to-use online services. Consequently, as users perceive the ease of use and the intuitiveness of the AI systems developed by companies, they become more conscious they have to trust and use them [60].

Also, this research pointed out that PC positively impacts AI. This significance supports hypothesis H4 and corroborates the studies of Esmaeilzadeh [61], Zhou, Liu, Yu, Tao, and Shao [63] and Wang, Ahmad, Bani Ahmad Ayassrah, Awwad, Irshad, Ali, Al-Razgan, Khan, and Han [62]. AI can provide different experiences to users, so users’ attitude toward AI technologies depends on the system models, consistency, and outcomes. Furthermore, AI control must be agile, reliable, and easy to perceive. Hence, users’ attitudes towards AI are influenced by their perceived control of AI systems [62]. As a result, users feel more confident and more willing to explore AI functionalities. Also, users’ attitudes toward AI tend to be influenced by their perception of its performance. Thus, the better the perceived control of users about AI, the better the attitude of users toward AI technologies, which is in accord with the TPB [62].

Regarding the AI construct, our results showed a significant impact on its interaction with PI. These empirical results validated hypothesis H5 and are in accord with prior studies about technology acceptance (TAM) [37,64,66] and AI impact on purchase intention [37,64,66]. Hence, our study reinforces the existing conception of the important role that AI plays in consumer decision-making [37,64,66]. AI is an important tool that urges consumers to search, compare, and purchase products online, which can offer consumers a great experience throughout the online purchase process. Nevertheless, AI must be convenient, intuitive, effectiveness, and easy to use [28,37,64,66], to promote satisfaction and the reduction of effort in order to generate a positive response from consumers towards the AI experience. Furthermore, the enjoyable interaction with AI creates more willingness and preference in consumers to engage in e-retail transactions.

Lastly, our results showed the mediator role that AI plays in the interaction mechanisms between the psychosocial factors analyzed and PI dependent variable, despite no significance being found in the direct relationship between SN and AI. This evidence supports hypotheses H6, H7, H8, and H9 and complements the knowledge about the mediator effects in PI [32,66] by suggesting that AI can also impact PI through its indirect effect. In particular, it is interesting to verify that PI activates the influence of SN on AI through a mediating effect when it comes to online retail. Therefore, the community (friends, family, general society) norms and expectations can positively influence the user’s attitudes towards AI technologies to help their PI when it comes to e-retail. These results supported the TPB [32,66] and the TAM [32,66] by demonstrating a relationship between user behavior and technology acceptance of user intention—all the psychosocial factors analyzed predict AI when AI is adopted by users to purchase intentions.

In summary, our findings relate that AI is perceived favorably by users when it is associated with purchase intentions in online retail. AI plays an important mediator role in the interaction mechanisms between the psychosocial factors in PI—AI indirectly links all the interaction mechanisms—as demonstrated through the significant effects of the endogenous variables. Regarding PI, this dependent variable stimulates and activates the SN effect on AI, which demonstrates that SN predicts AI when it comes to e-retailing.

### 5.2. Theoretical Implications

This study has several theoretical implications, namely for the Theory of Planned Behavior (TPB) and for the Technology Acceptance Model (TAM).

First, the research model proposed in this study provides a systematized perspective about the influencers of PI and extends the knowledge about the impact of AI in e-retail, which can help academics understand the role of AI technologies in consumer purchase intentions. It has validated that AI directly affects PI and simultaneously plays a mediator role in the interaction between the psychosocial factors analyzed and PI. Due to this evidence, AI can stimulate consumer engagement [70] and constitutes an alternative channel where consumers can be informed about their shopping alternatives.

Second, this study examined the effect of the interactions between AI and psychosocial factors in online retail to understand users’ behavior through the interplay and the role of each psychosocial factor. It was observed that the psychosocial factors have different impacts on the interaction with AI. The research model tested validated that C has a great impact on AI. This significance highlights the important role of confidence and satisfaction in adopting AI [60]. Hence, the results give insights to AI researchers and designers, suggesting that AI technologies should be efficient but also intuitive, easy to use, and enjoyable to generate confidence among users.

This study contributes a perspective on the influence of SN on AI, demonstrating that SN predicts AI when it comes to decisions related to purchases in online retail. This perspective enriches the literature about the relationship between subjective norms (one component of TPB) and TAM [25,28,46], evidenced by the fact that SN influences AI when the latter is adopted to help consumers in their PI online retail. Furthermore, this significant evidence demonstrated that the positive interaction between SN and IA does not occur in a generalized way.

Fourth, by exploring the significant mediator role of AI, this study validates that greater consumer satisfaction, confidence, and consciousness about the adoption of AI technologies implies greater willingness to adopt AI in e-retail [45,54,57]. Therefore, the high capacity of AI technologies to stimulate psychosocial factors can drive consumers’ PI and promote the continuous adoption of AI in e-retail when the experience of the consumers with AI is great. Thus, the combined approach of the TPB and TAM can provide insights into the multifaceted role that AI plays in e-retail by explaining the users’ behavior and influencing the users’ purchase decisions.

Finally, this research provides a reference about the influence of AI in e-retail marketing, suggesting that AI technologies are a tremendous opportunity for companies to improve engagement with consumers [2,66]. This implies that through AI technologies, companies can take the opportunity to build strong and effective relationships with consumers, which may increase satisfaction, loyalty, and sales.

### 5.3. Practical Implications

This study has several practical implications for academics, managers, and consumers. It addresses the main determinants that affect purchase decisions through the adoption of AI technologies.

First, this study has implications for academics and researchers by giving insights into the different influences of the psychosocial factors in AI and the role of AI in PI to online retail. Hence, the findings of this study support academics in exploring how the fundamental psychosocial factors influence customers’ behavior in their online shopping decisions. AI appears to be an effective and significant influencer of PI [64,66], but with different predicted effects. Consequently, academics can explore how psychosocial factors can influence the adoption of AI and how AI technologies will impact e-retail in the next years. Based on these focused areas, academics can contribute to the development of AI technologies according to consumers’ expectations and understand how different commercial activity sectors will adapt their marketing strategies to the democratized trend of e-commerce. This fact can contribute to enhancing technology acceptance.

Second, this study has several implications for companies. On the one hand, it provides insights into the main factors that affect the decisions of consumers regarding the use of AI in online shopping decisions. This contributes to helping retail managers in the choice of more efficient, confident, and enjoyable AI tools to stimulate the willingness of consumers to use them. Thus, managers can understand how AI technologies can be optimized for suitable consumer experiences and long-term customer engagement [21,22]. From another point of view, the adoption of AI in online shopping can help managers in the definition of personalized marketing strategies, based on the AI analysis derived from the consumer’s interaction with companies, and thereby in the definition of persuasive marketing strategies to influence their purchase decisions [3,4]. So, companies can use digital marketing strategies applied to AI to recognize and satisfy consumers’ needs and simultaneously encourage consumers to make purchase decisions. Additionally, AI tools help managers define the behavior and the profile of the consumers to adapt e-commerce strategies better. Hence, managers can increase business efficiency by developing personalized marketing actions [2] according to the recognized characteristics and preferences of the target, which can increase the possibility of more continuous purchases being made online.

Finally, this study has implications for the daily life of consumers and their purchase behavior. The impact of AI on consumers to help their PI is quite wide. AI facilitates interaction with businesses and promotes faster decision-making processes. Accordingly, consumers tend to be oriented to AI practices that empower decision-making with less effort and time spent and with high simplicity and convenience [32,38,41]. Also, AI tools enhance the shopping experience and influence the consumer’s behavior by providing several facilitated processes that can help consumers in their decision-making. This implies less effort and cost savings for consumers, which, in fact, stimulates the adoption of AI technologies to PI when it comes to e-retail. However, as AI technologies continue to evolve, their impact on e-retail will grow too, making it crucial that consumers stay informed about new AI tools developments.

### 5.4. Limitations and Future Lines of Research

Although this study offers important insights into how psychosocial factors influence AI and their impact on PI in e-retailing, it has some limitations.

Firstly, the results of this study cannot be generalized because they represent only part of the population. Thus, other studies could verify if these results can be replicated in other sociodemographic realities and analyze (possible) cultural implications in the acceptance of AI. Moreover, other studies can be performed using different samples or methods of data collection to combat possible sample biases that can be derived from data collected through online platforms. In this context, the response accuracy could be affected by desirability bias, rememberability bias, and honesty bias.

Another limitation is that this study is only focused on the perspective of consumers. Hence, subsequent research could extend the perspective of analysis to companies using a business-to-business approach. In this context, future researchers could analyze how AI affects purchase intention in different industries or, according to companies’ size, offer specific insights about different industry realities.

This study used a quantitative method, which is a common methodology used in most social and marketing research. However, this method does not recognize the importance of the context of the study and neglects the impact of the subjective interpretation of data, for example, their causality (the specific way in which the variables are related). Thus, future research could be performed through qualitative methods, such as in-depth interviews or case studies, to provide more in-depth insights about the psychosocial factors that affect AI and its cause–effect relationship with purchase intention based on an interpretative and exploratory approach to yield descriptive scenarios.

Another notable limitation is that this study does not consider the type of technologies employed in a relationship between AI and PI in online retail. AI technology is constantly evolving, which affects the dynamics of society’s general behavior, specifically consumers’ behavior. Hence, subsequent studies can assess how new and different AI technology applications and tools can affect the influence of PI.

## 6. Conclusions

The influence of AI on business has been noted in recent years, especially in e-commerce, which can represent a great opportunity to improve purchase intention. Furthermore, the adoption of AI technologies in e-retailing can improve consumers’ engagement with companies. In this context, this current study analyzed the influence of a set of psychosocial factors on the practical applicability of artificial intelligence-enabled ease of usage (AI) and its impact on purchase intention (PI) in online retail.

The research model proposed in this study showed statistical significance in the hypotheses tested. It has been shown that faith (F), consciousness (C), perceived control (PC), and subjective norms (SN), the latter observed in e-retail, positively impact AI, which in turn positively influences PI in online shopping. These observations allow us to conclude that the behavioral intentions in AI usage in e-retail can be predicted by integrating various psychosocial factors according to the TPB and the TAM.

From these results, it has been noted that faith stimulates the user’s attitude towards AI. Due to this evidence, confidence, beliefs, and benefits stimulate and motivate users to opt for AI when it is desired for virtual experiences. Furthermore, AI improves the preferences of users and increases their faith when providing suitable virtual experiences. On the other hand, it has been found that the consciousness of the users and their knowledge about virtual technologies encourage users to opt for AI experiences. Thus, users who feel safer and confident with virtual tools are more willing to opt for AI. Moreover, users are willing to adopt AI technologies when they are “easy-perceive” about their functionality, performance, and outcomes. Therefore, the user’s perception and control of AI technologies seem to be determinants of the user’s attitudes towards AI, which is in line with the TPB.

Next, the empirical results indicate a direct and indirect impact of AI in PI when it comes to e-retailing. AI appears to be an effective predictor of PI, which means that AI has a strong role in consumer behavior. This means that consumers feel motivated and willing to opt for AI to assist its PI in e-retailing. On the other hand, AI technologies may encourage consumers to carry out different actions during their PI, like search, compare, and simulation, which can contribute to increasing and extending the connections with companies and stimulating the interest in consumption. Thus, AI technologies have a multifaceted impact on consumers’ behavior when it comes to online retailing and can constitute a great opportunity for companies to enhance their engagement with consumers.

From another point of view, this study allows us to observe the important mediator role that AI plays in the interaction mechanisms between the four psychosocial factors considered—SN, F, C, and PC—and PI. In particular, it has validated that SN predicts AI when it comes to PI e-retailing. So, the acceptance of users of AI technologies is influenced by the experiences of friends, family, and the general community about PI in online retail. SN is an effective and persuasive factor. Therefore, the suitable experiences of the society associated with AI have a persuasive effect on the consciousness of users, which seems to motivate them to adopt AI in e-retailing.

In summary, this study demonstrates that AI influences consumer behavior and purchase intention. AI can provide several suitable experiences to consumers, like searching, comparing, and seeking in-depth characteristics about products and companies. Therefore, AI acceptance stimulates the willingness of consumers to make purchases because it provides a variety of abilities and facilitated interactions that can help and encourage their PI. This evidence lends support to the TPB and TAM. Otherwise, this study showed that AI contributes to extending the long-term relationship between consumers and companies, which turns AI into a great “partner” to companies, enhancing engagement with consumers and increasing business.

## Figures and Tables

**Table 1:** Instruments and definitions.

Instruments	Definition	Previous Studies
Subjective norms (SN)	Perceived social pressures to perform or not perform a particular behavior.	Ajzen [25], Chan and Wong [75]
Faith (F)	Form of basic knowledge that is intertwined with a certainty that leaves no room for doubt.	Davis [76], Gefen et al. [77], and Park [78]
Consciousness (C)	Refers to the state or quality of being aware of an external object or something within oneself. It involves the ability to perceive, experience, and have subjective experiences, such as thoughts, feelings, and sensations. Consciousness is a complex and multifaceted phenomenon that plays a crucial role in human cognition, perception, and self-awareness.	Bhagat, Chauhan, and Bhagat [41], Davis [76], Gefen, Karahanna, and Straub [77], and Park [78]
Perceived control (PC)	Refers to an individual’s belief or perception regarding their ability to influence or control environmental cues, stimuli, or outcomes in a given situation.	Ajzen [25] and Kautish and Khare [79]
AI-enabled ease of use	Refers to the integration of artificial intelligence technologies and capabilities into products, services, or systems to enhance user experience, simplify interactions, and streamline processes.	Lam et al. [80] and Parasuraman and Colby [81]
Purchase intention (PI)	Refers to a consumer’s willingness, inclination, or plan to buy a particular product or service in the future. It reflects the individual’s mindset and readiness to make a purchase decision, indicating the likelihood of converting interest or desire into an actual buying action.	Vermeir and Verbeke [82], Konuk [83], and Yadav and Pathak [84]

**Table 2:** Descriptive statistics, reliability, and validity for constructs.

Items	*M*	*SD*	Standardized Factor Loadings	*α*	CR	AVE
SN_1	3.66	1.69	0.84	0.82	0.83	0.54
SN_2	3.70	1.63	0.78			
SN_3	3.90	1.75	0.74			
SN_4	4.04	1.70	0.57			
F_1	4.08	1.62	0.80	0.82	0.82	0.60
F_2	4.32	1.59	0.77			
F_3	4.05	1.59	0.76			
C_1	4.26	1.71	0.78	0.69	0.70	0.64
C_2	4.53	1.62	0.63			
C_3	4.44	1.53	0.58			
PC_1	4.34	1.52	0.63	0.57	0.76	0.39
PC_2	4.24	1.58	0.82			
PC_3	3.75	1.57	0.70			
AI_1	4.55	1.60	0.70	0.84	0.80	0.64
AI_2	4.55	1.56	0.82			
AI_3	4.47	1.55	0.74			
PI_1	3.89	1.69	0.82	0.81	0.81	0.59
PI_2	3.99	1.63	0.76			
PI_3	3.59	1.75	0.71			

**Table 3:** Correlations matrix.

	SN	F	C	PC	PI	AI	VIF
SN							1.88
F	0.651 **						2.26
C	0.515 **	0.629 **					1.82
PC	0.513 **	0.541 **	0.525 **				1.61
PI	0.641 **	0.669 **	0.551 **	0.564 **		.	
AI	0.514 **	0.671 **	0.687 **	0.527 **	0.609 **		

** *p* < 0.001.

**Table 4:** Multiple linear regression analysis of predictors of artificial intelligence-enabled ease of use.

Predictors	*r*	*R*^2^ Adjusted	*F*	*p*	ß	*t*	*p*	*d*	95% Confidence Interval	
									Lower	Upper
Model	0.76	0.58	489.44	<0.001						
SN					0.02	1.10	0.271	0.000	−0.015	0.054
F					**0.34**	**13.12**	**<0.001**	0.128	0.286	0.387
C					**0.40**	**17.08**	**<0.001**	0.184	0.373	0.470
PC					**0.12**	**5.61**	**<0.001**	0.014	0.094	0.196

Note: Bold values show the presence of statistical significance.

**Table 5:** Multiple linear regression analysis of predictor of purchase intention.

Predictors	*r*	*R*^2^ Adjusted	*F*	*p*	ß	*t*	*p*	*d*	95% Confidence Interval	
									Lower	Upper
Model	0.61	0.37	845.65	<0.001						
AI					**0.61**	**29.08**	**<0.001**	0.59	0.601	0.687

Note: Bold value shows the presence of statistical significance.

**Table 6:** Indirect effects on endogenous variables.

Indirect Effects on Endogenous Variables	Path *(β)*	*t* Value (Bootstrap)	*p*-Value	95% Confidence Interval		Hypothesis Support
				Lower	Upper	
SN→AI→PI	0.195	20.840	<0.001	0.165	0.227	Yes
F→AI→PI	0.195	18.679	<0.001	0.153	0.237	Yes
C→AI→PI	0.299	8.995	<0.001	0.250	0.348	Yes
PC→AI→PI	0.227	14.622	<0.001	0.194	0.260	Yes

## Data Availability

The data presented in this study are available on request from the corresponding author. The data are not publicly available due to ethical and privacy considerations to protect the confidentiality of participants.
